# The effects of varying doses of caffeine on cardiac parasympathetic reactivation following an acute bout of anaerobic exercise in recreational athletes

**DOI:** 10.1186/s12970-020-00373-6

**Published:** 2020-08-20

**Authors:** Amir Sarshin, Alireza Naderi, Carlos Janssen Gomes da Cruz, Foad Feizolahi, Scott C. Forbes, Darren G. Candow, Ebrahim Mohammadgholian, Mehrdad Amiri, Naghmeh Jafari, Alireza Rahimi, Eidi Alijani, Conrad P. Earnest

**Affiliations:** 1 Department of Exercise Physiology, Karaj Branch, Islamic Azad University, Karaj, Iran; 2 Department of Sport Physiology, Boroujerd Branch, Islamic Azad University, Boroujerd, Iran; 3 Laboratory of Exercise Physiology, Faculty of Physical Education, University of Brasilia, Brasília, Brazil; 4 Faculty of Education, Department of Physical Education, Brandon University, Brandon, MB R7A6A9 Canada; 5 Faculty of Kinesiology and Health Studies, University of Regina, Regina, SK S4S0A2 Canada; 6 Health and Kinesiology, Texas A & M University, College Station, TX USA

**Keywords:** Exercise, Caffeine, Anaerobic performance, Dietary supplement, Heart rate variability, Autonomic balance

## Abstract

**Background:**

To examine the effects of varying doses of caffeine on autonomic reactivation following anaerobic exercise.

**Methods:**

Recreationally active males (*N* = 20; 24 ± 2y) participated in a randomized, double-blind, placebo-controlled, crossover study where participants ingested: [1] Control (CON; no supplement), [2] a non-caffeinated placebo (PLA), [3] 3-mg∙kg^− 1^ of caffeine (CAF3) or [4] 6-mg∙kg^− 1^ of caffeine (CAF6) prior to Wingate testing. Parasympathetic (lnRMSSD, primary outcome) and global HRV (lnSDNN, secondary outcome) were assessed at rest (i.e., pre-ingestion), 45-min post-ingestion, and 5-min and 35-min post-exercise recovery. We used a GLM to assess mean (95% CI) changes from pre-ingestion baseline.

**Results:**

Overall, we observed a significant trend for lnRMSSD and lnSDNN (*both*, *p* = 0.001, ηp^2^ = 0.745). Forty-five minutes after treatment ingestion, we observed a significant increase in lnRMSSD for CAF3 (0.15 ms, 95%CI, 0.07,0.24) and CAF6 (0.16 ms, 95%CI, 0.06,0.25), both being significant (*both*, *p* <  0.004) vs. CON (− 0.02 ms, 95%CI, − 0.09,0.04). Five-minutes after exercise, all treatments demonstrated significant declines in lnRMSSD vs. baseline (*all*, *p* <  0.001). After 35-min of recovery, lnRMSSD returned to a level not significantly different than baseline for CAF3 (0.03 ms, 95%CI, − 0.05, 0.12) and CAF6 (− 0.03 ms, 95%CI, − 0.17, 0.10), while PLA (− 0.16 ms, 95%CI, − 0.25, − 0.06) and CON (− 0.17 ms, 95%CI, − 0.28, − 0.07) treatments remained significantly depressed. A similar pattern was also observed for SDNN.

**Conclusion:**

Caffeine ingestion increases resting cardiac autonomic modulation and accelerates post-exercise autonomic recovery after a bout of anaerobic exercise in recreationally active young men. However, no differences between caffeine doses on cardiac autonomic reactivity were observed.

## Introduction

Adaptations to exercise training require an appropriate training stimulus accompanied by sufficient recovery [1]. One factor associated with exercise training and recovery is the balance between the sympathetic and parasympathetic nervous branches of the autonomic nervous system (ANS) [2]. Accordingly, an imbalance in ANS between the training stimuli and recovery can may lead to ANS dysregulation and negatively impact exercise performance [1, 3]. Fortunately, ANS can be assessed easily and non-invasively assess heart rate variability (HRV) via heart rate monitors, thus providing for a useful tool for sports performance and recovery from strenuous exercise.

During exercise, heart rate increases via parasympathetic withdrawal and increased sympathetic activity [2]. HRV becomes useful during training and as it a validated tool to assess internal training load during and following exercise allowing for the individual evaluation surrounding training stimuli [1, 3, 4]. Despite the potential implications of HRV, the time course of HRV following exercise is multifactorial and varies based on genetics, and the intensity, duration, and mode of exercise [2, 4–6]. Accordingly, exercise intensity seems to play the greatest role relative an athlete during acute post exercise HRV recovery, in particular parasympathetic reactivation, is delayed following high intensity exercise [4, 5, 7]. A potential, yet relatively unexplored means of improving parasympathetic activity following strenuous exercise is the ingestion of caffeine.

Caffeine is one of the most popular ergogenic compounds used in sport and supported by a large body of scientific evidence for improving anaerobic and aerobic activities [8–11]. The ingestion of caffeine influences the ANS via an increase in catecholamine secretion, which subsequently increases heart rate and mean arterial blood pressure at rest and during exercise [12]. Caffeine acts as a sympathetic stimulus during exercise and has been shown to attenuate autonomic recovery post-exercise [13, 14] . However, research findings are mixed, with some studies showing that 300–400 mg of caffeine delays post-exercise parasympathetic reactivation [12, 14, 15], while others have found no effect from caffeine at doses associated with < 3 mg∙kg^− 1^ body mass [16, 17]. While it is difficult to compare and contrast results across studies, these inconsistent findings may be related to the caffeine dosage used, which warrants further investigation within athletic populations. The purpose of this study was to examine the effects of two dosages of caffeine (3 mg∙kg^− 1^ and 6 mg∙kg^− 1^) on HRV indices of the ANS following a single bout of high intensity exercise. The primary outcome measure was root mean square of the successive differences (rMSSD), a time domain HRV index reflecting parasympathetic tone. The secondary outcome for our study was the standard deviation of the NN (R-R) intervals (SDNN). We hypothesized that caffeine ingestion would reactivate parasympathetic markers following a reduction immediately following exercise.

## Methods

### Participants

We enrolled healthy, recreationally active males (*N* = 20, age: 24 ± 2 y; body mass: 74.70 ± 7.07 kg; height: 178.8 ± 4.64 cm) who engaged in ≥3 sessions per week or 200–300 min∙wk.^− 1^ of exercise into the study. Inclusion criteria required participants to have been physically active for the 6-months preceding the study and not habitually consuming caffeine (< 120 mg∙day^− 1^; < 3 days/wk). All participants signed an informed consent following verbal and written information of the study design and potential risks before beginning the study. A health history questionnaire (HHQ) and physical activity readiness questionnaire (PAR-Q) were administered in order to ensure participants were eligible to do high intensity physical activity safely [18]. Individuals having had orthopedic complications or cardiovascular, pulmonary, or metabolic disease were excluded from the study. For all testing sessions, participants wore light and comfortable clothing, avoided strenuous exercise for 48 h and caffeine for 24 h. The study was approved by the Ethics Committee of Islamic Azad University of Karaj.

### Experimental design

The study used a double-blind, placebo-controlled, randomized cross-over design. An independent investigator not involved in data collection performed randomization and supplementation preparation. Participants visited the laboratory on six separate occasions within a 20-day period, with a minimum of 72 h between visits. During the first visit, the study procedures were explained, informed consent was obtained, and participants completed various questionnaires, such as the PAR-Q and HHQ. Participants also recorded their dietary intake prior to the first experimental day and were asked to replicate this diet on subsequent visits. Height (cm) was measured with an electronic stadiometer (SECA 217, Seca Ltd., Hamburg, Germany) to the nearest 0.01 cm without shoes and with each participant standing erect against a wall and body mass (kg) was measured to the nearest 0.01 kg using a calibrated digital scale (Seca 770-floor, Seca Ltd., Hamburg, Germany). During the second visit participants completed a 30-s all-out Wingate Anaerobic Test (WAnT) for familiarization and to reduce the learning effect [19, 20].

**Fig. 1:**
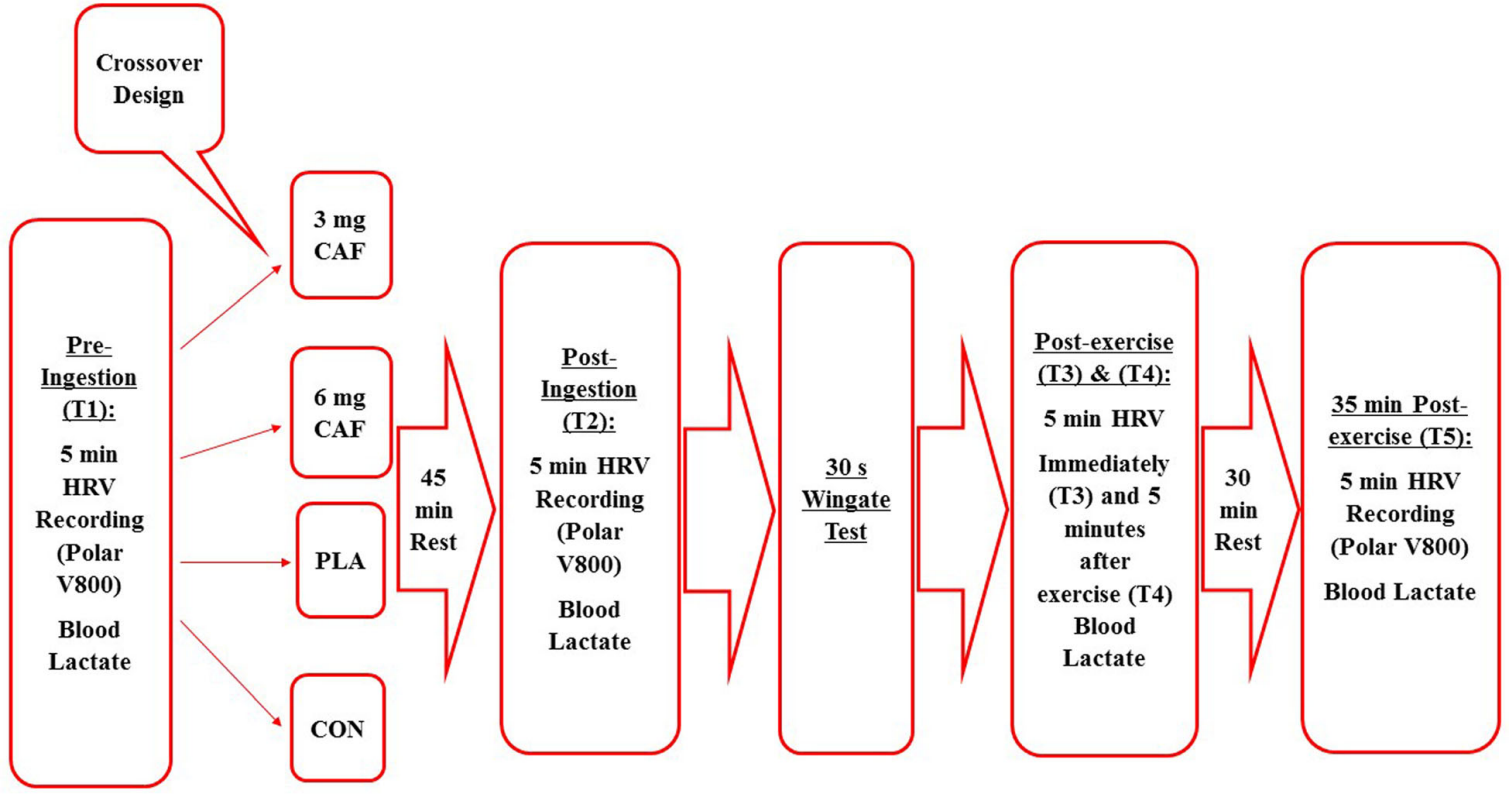
Schematic representation of the study procedures

During the remaining experimental visits [3–6], participants performed the experimental treatments based on computer generated randomization of four treatments: [1] control (no treatment), [2] placebo (PLA, non-caffeinated treatment matched for taste and color), [3] 3 mg∙kg^− 1^ of caffeine (CAF3) and [4] 6 mg∙kg^− 1^ of caffeine (CAF6). All experimental visits were conducted at the same time of day (between 11:00 and 14:30). To minimize potential gastrointestinal distress, participants consumed a standardized snack (white bread and boiled eggs) containing 3 g∙kg^− 1^ of carbohydrates, 20 g of protein, and 10 g fat 180–240 min before each session. A schematic of the testing protocol is presented in Fig. 1 and is detailed below:

- **(Time 1) Resting:** ***Pre-Ingestion***. Prior to treatment ingestion, participants sat quietly for 5-min before providing a resting blood sample to assess blood lactate (BLa; Lactate Scout plus analyzer, SensLab GmbH, Germany). A resting HRV was also measured. Following these baseline measurements participants then ingested their respective treatments.
- **(Time 2) Resting:** ***45-min Post Ingestion***. Forty-five minutes after treatment ingestion, participants were assessed again for HRV and BLa. Following this assessment, participants engaged in a standardized warm-up before performing WAnT testing (*detailed below*).
- **(Time 3) Post Exercise:** ***5-min Post WAnT***. Following their WAnT, participants were tested immediately for BLa, ratings of perceived exertion (RPE), and another HRV measurement was completed 5-min after exercise.
- **(Time 4) Post Exercise:** ***35-min post WAnT***. Following the 5-min post exercise assessment, participants engaged in a passive recovery period, and were subsequently assessed a final time for HRV and BLa 35 min post exercise.

### Wingate anaerobic test

Participants performed a Wingate Anaerobic Test (WAnT) using a friction-loaded cycle ergometer (MONARK 894E, Stockholm, Sweden) connected to a computer as previously described [19–21]. The ergometer was calibrated before each test. Briefly, the WAnT was a 30-s test requiring participants to pedal as fast as possible against a fixed resistance. Each test began with a 4-min standardized warm-up against a fixed load of 1 kilopond and three separate 2 s sprints were performed against a load of 0.075 kp∙kg^− 1^ of body mass were interspersed with 45-s of active recovery [20]. After the warm-up, one minute of light dynamic stretching was performed. The test protocol began with a verbal command of “faster, faster, 3,2,1, go”, during which the load of 0.075 kp∙kg^− 1^ body mass [22] was applied. At “go” the participants were pedaling as fast as possible and each participant was verbally encouraged to maintain as high of a cadence as possible over the entire 30-s. No visual or verbal feedback regarding the time to complete the test was provided.

Power output in watts was calculated as the product of resistance and flywheel revolutions, which was recorded every 1-s, peak power output was determined from the average of the first 5-s a mean power output was assessed as the average over the entire 30-s of the test. Fatigue index was calculated from the difference between peak 5 s power output and the power output that occurred during the final 5-s of the test divided by the peak power output multiplied by 100 [21]. The seat height and handlebars were adjusted such that the knee would be slightly bent at maximal leg extension and kept constant throughout the remaining experimental sessions.

### Supplementation protocol

Caffeine powder (Cat. No. C0750; Sigma Aldrich) and PLA (dextrose) treatments were packaged in identical gelatin capsules (Iran Gelatin Capsule Co. Iran). The capsules’ ingredients were unknown to participants and investigators who performed data collection. The PLA contained 200 mg dextrose, while the caffeine was provided at two doses: 3 mg∙kg^− 1^ of body mass or 6 mg∙kg^− 1^ of body mass.

### Heart rate variability measurement

Heart rate variability is a non-invasive tool used to quantify cardiovascular autonomic function based on the measurement of the timing between consecutive R-R intervals [23, 24]. Briefly, the heart rate monitor strap was placed on the participant according to the manufacture instructions. All HRV measurements were collected in a seated position, within a quiet and dimly lit room with no external stimuli. The R-R interval data were recorded at a sampling frequency of 1000 Hz for 5-min and was synchronized with the Polar Flow web service (Polar Flow software). Raw unfiltered R-R data was exported as a space delimitedtext file for analysis of time (Kubios V 2.2, Joensuu, Finland), as previously described [25]. Any segments that contained three or more irregular R-R intervals were excluded from analysis, and possible artifact noise was replaced with the interpolated adjacent R-R interval values (filter power < low). R-R interval markers were measured using a window width of 256-s and overlap of 50% through the specialized HRV software (Kubios V 2.2, Joensuu, Finland). The dependent variables in the time domain included the standard deviation of normal-to-normal (SDNN) intervals and the root mean squared of successive difference (RMSSD) of R-R intervals [26]. Log transformed data for all time were used in statistical analysis to avoid outliers.

### Statistical analysis

The primary outcome for our study was parasympathetic tone as measured via lnRMSSD as RMSSD is a singular index measuring parasympathetic nervous system tone. Our secondary outcome was lnSDNN, which reflects mixed ANS modulation and is considered a global index of HRV. We used general linear models to examine changes from the baseline, pre-ingestion HRV assessment at 45-min post-ingestion, 5-min post exercise and 45-min post exercise. Statistical analyses were performed using general linear models. Post-hoc analyses were performed using Bonferonni adjusted, paired t-tests in order to prevent the likelihood of Type I error (SPSS® version 25, IBM North America, New York, NY, USA). Normality was examined using Kolmogorov-Smirnov test and confirmed for all exercise related variables. However, we observed that HRV indices were not normally distributed. Therefore, we performed a natural log transformation for all HRV measures. Effect sizes are presented as partial Eta squared (ηp^2^) for the general linear models and Cohen’s D for simple effects. All calculations were accomplished using) and the probability level for statistical significance was pre-set at *P* = 0.05, while ES were calculated using means and pooled standard deviations (SD). Effect sizes for partial eta squared were interpreted as: 0.01, small; 0.06, medium and > .14, large [26]. Effect sizes for Cohen’s D were interpreted as 0.20 = small, 0.50 = moderate, 0.80 = large [27]. Data throughout the manuscript are presented as mean (SD) or mean change (95% CI).

## Results

**Fig. 2:**
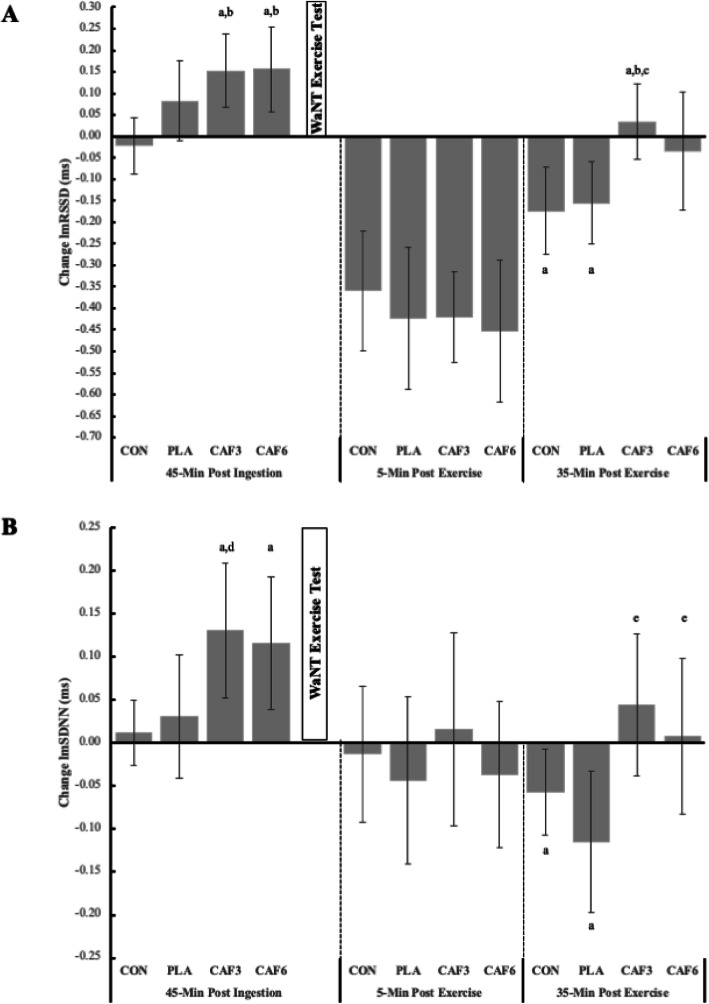
Data present change from baseline for lnRMSSD (panel **a**) and lnSDNN (panel **b**). Significance notations within each time period are as follows: (a) Significant vs. Baseline pre-ingestion (*p* < 0.001), (b) Significant vs. CON (*p* ≤ 0.004), (c) Significant vs. PLA (*p* = 0.011), (d) Significant vs. PLA and Control (*p* ≤ 0.033), and (e) Significant (*p* ≤ 0.003)

We have presented the results for WAnT testing in Table 1, inclusive of RPE and blood lactate. Further, we have presented a schematic representation of the study procedures in Fig. 1 and our findings for lnRMSSD and lnSDNN in Fig. 2. In brief, we observed that overall, participants generated a peak power output of 773.84 ± 178.41 W, a mean power of 502.96 ± 86.46, achieved a maximum exercise heart rate of 155.04 ± 7.36 b/min^− 1^, a maximal RPE of 16.37 ± 1.68, a maximal blood lactate level of 4.80 ± 0.65 mmol/L at peak exercise and 5-min post exercise 8.26 ± 1.08). Between group comparisons showed a significantly greater peak power output (157 W, 95% CI, 10.17, 304.42, *p* = 0.03), maximal HR (7.45 b/min^− 1^, 95% CI, 1.46, 13.44, *p* = 0.007). Both the CAF3 (− 1.80, 95% CI, − 3.05, − 0.55, *p* = 0.001) and CAF6 (− 2.25, 95% CI, − 3.60, − 1.10, *p* <  0.001) exhibited a lower maximal RPE vs. CON and PLA. No significant between group differences were observed for mean power, minimum power or fatigue index.

**Table 1:** Wingate test characteristics for the study participants (*N* = 20)

	All		CON		PLA		CAF3		CAF6		Sign.	ηp^**2**^	Effect
	Mean	SD	Mean	SD	Mean	SD	Mean	SD	Mean	SD			Level
**Peak Power (W)**	773.81	178.41	693.93b	159.16	749.80a,b	174.55	800.30a,b	174.04	851.22a,b	178.58	0.032	0.109	Medium
**Mean Power (W)**	502.96	86.46	473.21a	79.84	501.11a	91.11	510.14a	78.67	527.36a	92.76	0.252	0.052	Small
**Min Power (W)**	279.23	73.20	294.19a	66.04	262.39a	93.79	284.25a	64.18	276.09a	66.55	0.575	0.026	Small
**Fatigue Index**	62.02	12.92	55.70a	12.67	63.02a	15.53	63.02a	10.95	66.34a	10.43	0.061	0.092	Medium
**Max Heart Rate (b/min)**	155.50	7.59	151.30_a,b_	5.39	154.60_a_	8.06	155.50_b_	7.59	158.75_a,b_	6.65	0.013	0.131	Medium
**RPE Warm Up**	9.36	1.03	9.00_a_	0.80	9.30_a_	0.73	9.00_a_	1.35	9.00a	0.80	0.104	0.077	Small
**RPE Maximal Exercise**	16.37	1.68	17.70a	1.30	16.55a	1.79	15.90a	1.34	15.35a	1.34	< 0.001	0.275	Large
**Blood Lactate**													
**Pre-Ingestion**	1.19	0.1	1.19a	0.11	1.20a	0.1	1.17a	0.08	1.20a	0.09	0.725	0.017	Small
**45-Min Post Ingestion**	1.22	0.11	1.21a	0.13	1.18a	0.06	1.25a	0.09	1.22a	0.12	0.291	0.048	Small
**Maximal Exercise**	4.8	0.65	4.80a	0.67	4.90a	0.69	4.58a	0.48	4.94a	0.74	0.291	0.048	Small
**5-min Post Exercise**	8.26	1.08	8.18c	0.77	8.63b,c	0.97	7.13a	0.84	9.10b	0.56	< 0.001	0.469	Large
**35-Min Post Exercise**	2.93	0.43	2.86a,b	0.50	2.77b	0.32	2.90a,b	0.35	3.19a	0.45	0.013	0.131	Medium

Values in the same row and sub-table not sharing the same subscript are significantly different at *p* < 0.05 in the two-sided test of equality for column means. Tests are adjusted for all pairwise comparisons within each row using a Bonferroni correction

### Primary outcome: lnRMSSD

We observed a significant trend for changes in lnRMSSD (Fig. 2a) and lnSDNN (Fig. 2b), from baseline, following treatment ingestion (*both*, *p =* 0.001, ηp^2^ = 0.745). For lnRSSD, significant treatment effects were observed 45-min following ingestion (*p* <  0.009, ηp^2^ = 0.140) and 35-min post exercise (*p* = 0.014, ηp^2^ = 0.129). While all groups demonstrated a significant reduction in 5-min lnRMSSD vs. baseline (*all*, *p* < 0.001); no significant between group (i.e., treatment) were observed at this time (*p* = 0.809, ηp^2^ = 0.013). Specific between group comparisons findings demonstrated that the CAF3 and CAF6 treatments increased lnRMSSD significantly 45-min after treatment ingestion (*both*, *p <* 0.001) both CAF treatments to be significant vs. CON (*p* < 0.004). Thirty-five minutes after the completion exercise, both CAF3 and CAF6 treatments demonstrated a return of lnRMSSD to values not significantly different to baseline, while the CON and PLA treatments remained significantly depressed (*both*, *p* < 0.001). For this latter assessment, the CAF3 treatment was significantly higher than CON (*p* < 0.004) and PLA (*p* < 0.011) treatments (Fig. 2).

### Secondary outcome: lnSDNN

For lnSDNN, we observed significant treatment effects 45-min post-treatment ingestion (*p* < 0.023, ηp^2^ = 0.117) and 35-min post exercise performance (*p* = 0.017, ηp^2^ = 0.124), but not at 5-min post exercise (*p* = 0.784, ηp^2^ = 0.014), BMI 23.3 (SD). Forty-five minutes following treatment ingestion and before exercise testing, we observed a significant increase from baseline for lnSDNN in the CAF3 and CAF6 treatment conditions (*both*, *p* < 0.001). Both the CAF3 and CAF6 treatments were significant vs. the CON treatment (*p <* 0.004). Following WAnT testing, we observed a significant reduction in lnSDNN for all treatments after 5-min of recovery compared to baseline, pre-ingestion (*all*, *p <* 0.001). After 35-min of exercise, we observed a continued reduction in lnSDNN for the CON and PLA treatments (*both*, *p <* 0.001); however, no significant reductions in lnSDNN vs. baseline, pre-ingestion were noted for the CAF3 and CAF6 treatments. For this latter assessment, lnSDNN was significantly greater for the CAF3 and CAF6 treatments vs. PLA (*p <* 0.003). In summary, the HRV indices measured in this study returned to baseline conditions for both caffeine treatments, while the same indices remain reduced compared to baseline following a single bout of strenuous exercise.

## Discussion

The primary objective of this study was to analyze the effects of different caffeine dosages on resting and post-exercise cardiac autonomic modulation. While higher cardiac parasympathetic and global modulations were observed after CAF3 and CAF6 ingestion during the resting condition, no such effects were noted for the PLA and CON groups. Further, while all treatment groups demonstrated a significant reduction in lnRMSSD and lnSDNN 5-min following exercise, no between treatment effects were noted. Finally, given the continual HRV suppression for the PLA and CON groups at 35-min post-exercise, compared to the restoration of said indices for the CAF3 and CAF6 treatments to levels not significantly different from baseline, we conclude that the CAF ingestion in the quantities used in this study are sufficient to accelerates post-exercise autonomic recovery following a single bout of strenuous exercise. Based on these observations we accept our research hypothesis.

The effects of caffeine ingestion on resting HRV are conflicting, with studies reporting increases [28–30], reduction [31] and no changes [32] of resting parasympathetic and/or global modulation markers. Establishing a cause of these divergences is not an easy task since several variables can affect HRV analysis, such as sex [33], body position [34], body mass index [35], nutritional status [36], functional condition [37], corresponding heart rate [38], cardiorespiratory fitness [39] and age [40]. In that same sense, the physiological and functional response to caffeine ingestion also depends on various factors such as individual caffeine habituation [41], caffeine dosage [42], sex [43], functional condition adopted to analysis [32], genetic profile [44], caffeine expectancies [45] and some other neuromuscular characteristics [46]. Thus, it is plausible to infer that the autonomic response to caffeine ingestion is dependent on several independent variables, and the increase of cardiac parasympathetic and global modulations observed in this study may be limited to our study design and participants’ characteristics.

Regarding the caffeine dosage effect, no differences between CAF3 and CAF6 on resting autonomic dynamics were observed by this study. Previous studies showed that both 2 [28] and 5 mg∙kg^− 1^ [30] of caffeine, dosage close to those adopted in this study, were able to increase cardiac parasympathetic modulation. In this scenario, our results reinforce the possibility of increasing parasympathetic modulation after caffeine intake and add important information suggesting that the relationship between caffeine dosage and parasympathetic reactivity is not linear. No changes on lnRMSSD and lnSDNN were observed after 45 min of resting on PLA and CON groups, suggesting no effect of resting time on rest cardiac autonomic modulation. In opposition, a significant effect of resting time on supine and orthostatic cardiac parasympathetic and global modulation was previously observed after 60 min of resting in the supine position in young men [32]. In this same scenario, Zimmermann-Viehoff et al. (2015) observed a significant effect of resting time on HRV parameters in a sample of young men and women from 30 to 50 min of rest at the seated position [47] . Despite ours and Zimmermann-Viehoff et al. (2015) studies using the seated position to analyze HRV, some differences between them and may explain the conflicting results observed. These differences include the amplitude of R-R interval segments used for HRV analysis, rest time before the nutritional intervention and sample characteristics. Thus, these data indicate that the effect of resting time on HRV may be protocol-dependent and should be considered in studies involving the effect of different pharmacological and non-pharmacological interventions on HRV.

In the initial post-exercise analysis, all treatment groups demonstrated a significant reduction in lnRMSSD and lnSDNN, but no differences between treatments were noted. However, after 35 min of passive recovery no differences between rest and post-exercise lnRMSSD and lnSDNN were identified in CAF3 and CAF6 protocols, while a persistent depression of these autonomic markers was identified in CON and PLA groups. Thus, these results confirm our initial hypothesis (please, check it) that caffeine intake can boosts post-exercise cardiac autonomic recovery. Corroborating our results, Rolim et al. (2018) observed a higher post-exercise cardiac parasympathetic reactivation after a submaximal exercise test in young men after caffeine uptake (3 mg∙kg^− 1^), despite no changes in resting markers of cardiac autonomic modulation [32]. On the other hand, Kliszczewicz et al. (2018) underwent ten physically active young males to Wingate anaerobic test, and no effect of a complex containing caffeine (100 mg) + *Citrus aurantium* (100 mg) was observed on post-exercise parasympathetic and sympathetic activity, despite higher resting sympathetic activity compared to the placebo condition [17]. Otherwise, Bunsawat et al. (2014) suggest that caffeine can promote a sympathetic over activation after maximal exercise, a hypothesis based mainly on higher absolute heart rate and blood pressure after an exercise test [14]. However, higher training load and maximum heart rate were observed in Bunsawat’s study after caffeine intake, with no differences in heart rate recovery at the first and the second-minute post-exercise, which makes the caffeine-induced sympathetic over activation hypothesis questionable. In other words, higher post-exercise absolute heart rate and blood pressure may occur due to a higher training load and not necessarily a direct effect of caffeine ingestion.

From a physiological perspective, the autonomic response to caffeine uptake is complex and may be bilateral. Was previously reported that caffeine ingestion could promote a significant increase in plasma levels of catecholamines [48, 49] and inhibits the enzymatic degradation of cyclic adenosine monophosphate by phosphodiesterases, which potentiates postsynaptic neurotransmission in the sympathetic nervous system [50] On the other hand, despite parasympathetic response to caffeine uptake remain underexplored, it has been shown that caffeine can stimulate acetylcholine receptors and acts as an inhibitor of acetylcholinesterase [51, 52], which explains, at least in part, the caffeine-induced increase in parasympathetic activity reported in our and previous studies. In addition, it has been hypothesized that the caffeine-induced parasympathetic activation may be a result of baroreflex activation due to an increase in peripheral vascular resistance and blood pressure resulting from antagonistic caffeine effect on adenosine receptors [32], which need to be confirmed in future studies.

Notwithstanding a lack of difference between group mean power observed in caffeine and placebo protocols, higher peak power was observed in CAF3 and CAF6 compared to PLA and control reveal an ergogenic effect of caffeine on anaerobic performance. Also, higher peak power in CAF6 compared to CAF3 indicate that this ergogenic effect is dose dependent. Previously, a lack of effect and even reduction in anaerobic performance after caffeine consumption has already been reported in the literature [48]. However, a recent meta-analysis using studies of good and excellent methodological quality reveal that caffeine intake can augment mean and peak power output on the Wingate anaerobic test by 3 and 4%, respectively [11] . Interestingly, in our study, higher cardiac parasympathetic reactivation after caffeine intake was observed even in the face of higher peak power in CAF3 and CAF6 compared to control and PLA protocols. This finding strengthens the favorable effect of caffeine on post-exercise parasympathetic reactivation since an inverse relationship between exercise intensity and the magnitude of parasympathetic reactivation is expected [53, 54].

While a higher fatigue index was found following caffeine compared to control, but there were no differences compared to placebo. Of note, examining the effect of caffeine supplementation on repeated bouts of Wingate tests (four 30-s Wingate tests with 4 min of rest between each exercise) after caffeine (6 mg∙kg^− 1^) or placebo ingestion, Greer, McLean, and Graham (1998) observed that caffeine ingestion had an ergolytic effect in the latter two exercise bouts [48]. Otherwise, it was recently reported that caffeine supplementation (6 mg∙kg^− 1^) increased the peak power during Wingate anaerobic test and diminished neuromuscular fatigue, shown by attenuation of decrease in countermovement jump performance after Wingate test [55] . Thus, since increase [55, 56] and reduction [48, 57] of different markers of exercise tolerance after caffeine supplementation already been reported, the recommendation of caffeine supplementation to improve recreational or athletic performance should be made cautiously.

Despite no observed difference between RPE in caffeine and placebo during warm-up, the main effect of treatment and lower RPE observed after CAF6 compared to control and PLA indicates that caffeine may reduce the exercise-induced psychological stress. Interestingly, Duncan et al. (2019) observed a reduction of RPE during Wingate test for the upper-body, but not for the lower-body segment, suggesting that caffeine’s effect on RPE depends on body segment exercised [58]. Despite the absence of caffeine effect on RPE during lower-body Wingate test observed in some studies [58–60], our findings reveal that this benefit can be acquired with caffeine supplementation in this condition. We note that lower RPE identified in CAF6 protocol was accompanied by high peak power and mean power, which reinforce the psychostimulant effect of caffeine. It is an interesting approach since increases in exercise performance without altering RPE mean a higher power output without the increase in psychological stress per se; this positive effect should also be investigated in future studies.

As expected, an increase of BLA was observed after WAnT in all protocols indicating the vital contribution of anaerobic metabolism to the energy requirements during the exercise test. Despite increase [61] and maintenance [58] of BLA levels are commonly reported after caffeine intake, lower BLA concentration was observed in CAF3 compared to other protocols after five minutes of recovery. Unfortunately, the only lactate analysis performed in the initial phase of recovery does not permit to detect the exact moment with the highest lactate concentration, which makes any inference about the effect of caffeine on lactate production or clearance questionable. In the final phase of post-exercise recovery, we observed higher BLA levels in CAF6 compared to PLA, but the absence of difference between CAF6 and control prevents the attribution of higher blood lactate to caffeine supplementation. Of note, blood lactate reflects the balance between lactate production and clearance and the precise mechanisms that explain the small differences observed in this study is unclear and it may be just a inter day variation of BLA response to exercise, hypothesis previously reported in the literature [62, 63].

### Limitations

A major strength of our study is our randomized, crossover design. A limitation of our study is the use of a single, acute bout of WAnT testing. Therefore, we cannot generalize our findings to higher exercise volume conditions, such as multiple WAnT testing, multiple sets of resistance training, interval style workouts. We also cannot generalize our findings to women. The absence of ventilatory, sympathetic activity, and post-exercise blood pressure analysis, variables that influence HRV could also contribute to a better physiological interpretation of our data. We believe that a particular strength of our study was the use of a non-supplemented CON condition in addition an inert PLA and support this contention that a number of between group comparisons in our study were significant vs. the CON, but not the PLA treatments. Lastly, we assessed cardiac parasympathetic reactivation during 35 min of recovery, which limits our conclusions to this time window. However, despite the mentioned limitations, the analysis of autonomic response to different caffeine supplementation dosages on resting and post-exercise conditions adopted in this study adds robust information to current scientific debate about the autonomic effect of caffeine ingestion. The post-exercise time window adopted in this study allow fast and slow parasympathetic reactivation analysis and is within of window of opportunity for sudden death in young observed 30 min after vigorous exercise, which can be partially attributed to post-exercise cardiac autonomic dysfunction [64] and add clinical relevance to our results.

## Conclusion

We conclude that caffeine ingestion increases resting cardiac autonomic modulation and accelerates post-exercise autonomic recovery after a bout of anaerobic exercise in recreationally active young men. However, no differences between caffeine doses (3 or 6 mg∙kg^− 1^) on cardiac autonomic reactivity were observed.

## Data Availability

Data and publication materials are available from the corresponding author on reasonable request.

## Abbreviations

ACSM: American College of Sports Medicine
ANS: Autonomic nervous system
Bla: Blood lactic acid
CAF3: Caffeine treatment (3 mg/kg)
CAF6: Caffeine treatment (6 mg/kg)
CI: Confidence interval
CON: Control treatment
GLM: General linear model
HHQ: Health history questionnaire
hr.: Hour
HRV: Heart rate variability
kg: Kilogram
lnRMSSD: Log normalized root mean square of the successive differences
lnSDNN: Log normlized standard deviation of the NN (R-R) intervals
mg: Milligram
min: Minute
ms: Millisecond
NN (R-R): ECG intervals between R-waves
PAR-Q: Physical activity readiness questionnaire
PLA: Placebo treatment
RMSSD: Root mean square of the successive differences
RPE: Rate of Perceived Exertion
SD: Standard deviation
SDNN: Standard deviation of the NN (R-R) intervals
WAnT: Wingate anaerobic test
wk.: Week
